# Phytoestrogens as Natural Anti‐Aging Solutions for Enhanced Collagen Synthesis in Skin

**DOI:** 10.1111/jocd.16719

**Published:** 2024-12-09

**Authors:** Nahid Amini, Christina Osterlund, Jessen Curpen, Virginie Lafon‐Kolb, Thibaud Richard, Lene Visdal‐Johnsen

**Affiliations:** ^1^ Global Research and Development Oriflame Cosmetics Stockholm Sweden

**Keywords:** collagen, data mining, dermal density, *Glycyrrhiza uralensis*, phytoestrogen

## Abstract

**Background:**

The dermal extracellular matrix (ECM) is a dynamic scaffold composed mainly of proteins, with collagen as the key structural component providing resilience and support to the skin. Post‐menopause, declining estrogen levels lead to a significant reduction in skin health, notably a 30% decrease in collagen types I and III within 5 years.

**Aim:**

To discover natural extracts that stimulate collagen production.

**Methods:**

We utilized PathwayStudio to analyze protein–protein interactions and identify regulators of essential collagen types. Our study assessed *Glycyrrhiza uralensis* extract's ability to boost collagen production and enhance dermal density both in vitro and in vivo.

**Results:**

PathwayStudio analysis highlighted phytoestrogens including glycyrrhizin, isoliquiritigenin, liquiritigenin, liquiritin, and glabrol, as potential candidates. Liquorice rhizome (*G. uralensis*), used in traditional Chinese medicine, is rich in phytoestrogens like liquiritigenin. The *G. uralensis* extract increased collagen I and III gene expression and pro‐collagen I protein levels in human dermal fibroblasts and inhibited UVB and pollution‐induced matrix metalloproteinase‐1 (MMP1) production. In an in vivo study, a topical formulation containing the extract significantly improved dermal density after 56 days, measured by the DUB SkinScanner.

**Conclusions:**

These findings suggest *G. uralensis* extract as a promising agent for enhancing collagen production and skin health, particularly in postmenopausal women. Further research is needed to explore its mechanisms and long‐term effects.

## 1 Introduction

The dermal extracellular matrix (ECM) serves as a scaffold composed of various proteins, with collagen being the predominant component that imparts durability and structural support to the skin. The structural and functional significance of collagen in the skin cannot be overstated. Collagen fibers, primarily type I and III, confer tensile strength and contribute to the maintenance of skin thickness and elasticity. The delicate balance between collagen synthesis and degradation is paramount for skin homeostasis, and disruptions in this equilibrium are implicated in various dermatological conditions, including premature aging, skin sagging, and impaired wound healing [1].

Collagen synthesis, degradation, and remodeling are meticulously regulated processes, with hormonal signals playing a pivotal role in modulating these intricate mechanisms. Among these hormones, estrogen, traditionally linked to reproductive functions, emerges as a key influencer of skin physiology with its multifaceted effects. The decline in estrogen levels, particularly during menopause, represents a significant reduction in skin health such as overall thinning, fine wrinkles, and sagging, accompanied by various age‐related conditions and disorders [2]. Studies suggest a decline of up to 30% in types I and III collagen within the first 5 years post‐menopause [3]. Furthermore, estrogen is also strongly associated with mood and well‐being [4]. The diminishing levels of estrogen with age correlate strongly with the age‐related decline in muscle and bone mass and strength, ultimately impacting the human health span [5].

Numerous studies have demonstrated that hormone replacement therapy (HRT) involving estrogen can enhance skin collagen levels and thickness [6, 7]. Despite its promising effects, the long‐term use of HRT has raised concerns due to heightened risks of breast and total cancers [8, 9]. Consequently, there has been a shift towards exploring local topical hormone administration or newer cosmeceutical agents like selective estrogen receptor modulators (SERMs), including phytoestrogens, as appealing ingredients in skincare products, particularly for managing postmenopausal skin. This approach allows for a more targeted impact on the skin while mitigating potential adverse effects on the breast and uterus [10].

To identify novel natural extracts capable of stimulating collagen production, we employed a data mining approach. The scientific literature represents a vast repository of protein–protein interactions modulated by the skin exposome, with hundreds to thousands of such interactions reported annually in scientific journals. This abundance of data offers an opportunity to uncover associations between a target protein, its potential interaction partners, and the exposome such as air pollution or UV light. Utilizing tools such as PathwayStudio (Elsevier), one can effectively mine the literature to identify both positive and negative regulators upstream of key collagen types, including COL1A1, COL1A2 (collagen I), COL3A1 (collagen III), and COL4A1 (collagen IV). This approach led to the identification of several phytoestrogens such as glycyrrhizin, isoliquiritigenin, liquiritigenin, liquiritin, and glabrol, as promising candidates for future investigations.

Phytoestrogens are plant derivatives that structurally resemble endogenous estrogens and can be classified into three categories: isoflavones, coumestans, and lignans with isoflavones being the most widespread. Several studies have shown the beneficial effects of different phytoestrogens, such as genistein, daidzein, and equol on aging skin, including photoprotection, reduction of skin wrinkles enhancement of skin elasticity, and improvement in skin hydration [11]. Phytoestrogens, topical and systemic, appear to be an effective method in the treatment of intrinsic skin aging and are appealing ingredients in skincare products, particularly for managing postmenopausal skin.

Liquorice, a member of the *Glycyrrhiza* genus, is one of the most significant herbal medicines in traditional Chinese medicine, recognized as a source of the phytoestrogens liquiritigenin and its glycosides. The *Glycyrrhiza* genus, belonging to the *Leguminosae* family, encompasses over 30 species utilized in cosmeceuticals, containing numerous flavonoids primarily renowned for their whitening properties [12]. Several of these flavonoids also demonstrate estrogenic activity and extracts from various *Glycyrrhiza* species have historically been employed as botanical supplements to alleviate menopausal symptoms [13]. Among the three species of liquorice, namely *Glycyrrhiza uralensis*, *Glycyrrhiza glabra*, and *Glycyrrhiza inflate*, listed in the Chinese Pharmacopeia, the rhizomes, and roots of *G. uralensis* have been reported to possess the highest concentration of liquiritigenin and its glycosides [14, 15].

This paper presents the analysis and evaluation of the capacity of *G. uralensis* extract, assessed both in vitro and in a topical formulation in vivo, to induce collagen production and improve dermal density in the skin. Our study aims to explore the potential of *G. uralensis* as a natural alternative to estrogen replacement therapy in skin care, offering a promising topical solution to mitigate the adverse effects of estrogen decline on skin health and appearance.

## 2 Materials and Methods

### 2.1 Data Mining of Skin Exposome Dependent Protein–Protein Interactions

Literature data were evaluated and modeled into proteins (nodes) and their interactions for COL1A1, COL1A2, COL3A1, and COL4A2 in human cells and their interaction with the exposome (UV, ROS, diesel particulate matter (DPM)). The π–π interactions supported by less than three publications were excluded (Pathway Studio 11.4.0.8, Elsevier). Next, only interactions with proteins expressed in human skin tissues were kept, based on the Human Protein Atlas [16]. Finally, the 41 nodes and their interactions were loaded in Cytoscape 3.7.1, representing the final protein–protein network. However, to refine the data and pinpoint the most auspicious targets, it is necessary to apply relevant filters and visualization tools specialized in molecular interaction networks [17].

### 2.2 Extraction of Plant Material

Rhizomes of *G. uralensis* were dried, ground, and phytochemicals were extracted using the following method. Twenty milliliter of water was added to 1 g of plant material and placed in an ultrasonic bath (45 Hz, 5510 Branson) for 2 h at room temperature. The resulting suspension was centrifuged (5702 Eppendorf), filtered using 0.45 μm paper filter (Whatman), and freeze‐dried (FreeZone 4.5^Plus^, Labconco) for 48 h. The dried extract was analyzed using HPLC/UV and LC/QToF‐MS.

### 2.3 HPLC/UV Analysis

To quantify liquiritigenin in the extract, a 1 mg/mL solution of liquiritigenin (Sigma) was prepared and successively diluted in 95:5 v/v water:acetonitrile. A calibration curve was prepared using an Agilent 1260 Infinity System equipped with a Diode Array Detector (*λ* 280 nm). The column used was a reversed‐phase C18, 250 mm × 4.6 mm, 5 μm (Zorbax, Agilent) kept at 35°C and the mobile phase consisted of water (MilliQ) plus 0.1% of formic acid (A) and acetonitrile plus 0.1% of formic acid (B). The gradient started with 20% B and reached 60% after 10 min. The flow rate was 1.0 mL/min. The same instrumental setting was used to analyze liquiritin (Sigma) and liquiritin apioside (Sigma).

### 2.4 HPLC/QToF‐MS Analysis

The extract was analyzed by an Agilent 1200 series HPLC system coupled to an Agilent 6520 Q‐TOF mass detector. A C18 Zorbax column (250 mm × 4.6 mm, 5 μm, Agilent) kept at 35°C was employed for the separation of phytometabolites. The mobile phase consisted of water (MilliQ) plus 0.1% of formic acid (A) and acetonitrile plus 0.1% of formic acid (B) and the flow rate was set to 0.2 mL/min. The gradient started with 5% B and reached 40% B after 60 min and 95% B after 90 min. Mass spectral data were acquired in the 100–1000 m/z range, at a rate of 1.3 spectra/s. Ionization was achieved by an electrospray source in the negative mode (−ESI) using the following settings: drying gas flow rate 5 mL/min, temperature 325°C, nebulizer pressure 45 psi, and fragmentor 150 V. Data acquisition and processing were performed by Agilent Mass Hunter.

### 2.5 Viability Assay

In parallel with the other assays, viability was assessed using CellTiter‐Glo (Promega) following the manufacturer's guidelines.

### 2.6 Cell Culture

Primary human epidermal keratinocytes and dermal fibroblasts were sourced from healthy female donors aged between 24 and 56 years (CellSystems GmbH). They were maintained in EpiLife supplemented with HKGS (Gibco) or DMEM supplemented with 10% fetal bovine serum (Gibco), respectively. The cultures were incubated at 37°C in a humidified atmosphere containing 5% CO_2_.

### 2.7 UVB or Pollution‐Induced Keratinocyte/Fibroblast Assay

The UVB keratinocyte/fibroblast assay is a paracrine assay where media from UV‐stimulated primary keratinocytes activates primary dermal fibroblasts (method adapted from Wang et al. [18]).

In brief, primary keratinocytes were cultured until reaching approximately 80% confluence, followed by exposure to either UV radiation (50 mJ/cm^2^ within the UVB range of 280–315 nm using a UV‐MAT Irradiation controller [Dr Gröbel GmbH]) or pollution (DPM, 10 μg/mL [1650b, National Institute of Standards and Technology]). Subsequently, the cells were maintained at 37°C in a 5% CO_2_ humidified incubator, and the media was collected after 24 h.

Meanwhile, primary dermal fibroblasts were cultured until reaching approximately 80% confluence and pre‐treated with *G. uralensis* (equivalent to 5 μM liquiritigenin) for 1 h before exposure to the media from UV or pollution‐treated keratinocytes and freshly added *G. uralensis* extract (equivalent to 5 μM liquiritigenin). After 24 h of incubation, the media was collected and subjected to matrix metalloproteinase‐1 (MMP1) enzyme‐linked immunosorbent assay (ELISA, Sigma Aldrich) according to the manufacturer's instructions.

A standard curve was constructed from the ELISA data by plotting log concentration against absorbance (subtracting the blank) and subsequently analyzed using a variable slope four‐parameter curve fit.

### 2.8 Collagen Gene Expression Assay

To investigate the impact of *G. uralensis* extract on collagen I and III gene expression in primary dermal fibroblasts, quantitative polymerase chain reaction (qPCR) analysis was conducted.

Primary dermal fibroblasts were grown until they reached around 80% confluence. They were then exposed to *G. uralensis* extract (equivalent to 5 μM liquiritigenin) for 24 h before undergoing qPCR analysis.

### 2.9 qPCR Analysis

#### 2.9.1 RNA and cDNA

RNA extraction was carried out using the RNeasy Mini Kit (Qiagen), while reverse transcription of RNA was conducted utilizing the iScript Advanced cDNA Synthesis Kit (BioRad), following the guidelines provided by the respective manufacturers.

#### 2.9.2 qPCR Analysis

The real‐time PCR reaction was conducted in a total volume of 20 μL, comprising 10 μL of SsoAdvanced Universal SYBR Green Supermix (BioRad), cDNA, and the respective PrimePCR SYBR assays for *COL1A1*, *COL3A1*, *COL4A1* and *FBN* (BioRad). Thermal cycling was performed in a CFX96 ThermalCycler (BioRad) with a program consisting of initial denaturation at 95°C for 3 min, followed by 40 cycles involving denaturation at 95°C for 10 s, and annealing/elongation at 55°C for 30 s. Gene expression levels were normalized to the *GAPDH* housekeeping gene (PrimePCR SYBR GAPDH, BioRad). Relative changes in gene expression were calculated using the 2^−∆∆CT^ method and presented as fold changes compared to control samples.

### 2.10 Pro‐Collagen I Assay

Human primary fibroblasts were cultured until reaching approximately 80% confluence. Subsequently, the fibroblasts were subjected to treatment with *G. uralensis* extract (equivalent to 5 and 0.5 μM liquiritigenin) for a duration of 48 h in serum‐free media. Following treatment, the media was collected, and the levels of pro‐collagen I were quantified using an ELISA, in accordance with the manufacturer's protocol (Quidel). The pro‐collagen I data obtained from the ELISA analysis was then normalized to the total protein content determined using the bicinchoninic acid (BCA) assay (Sigma). The normalized data were compared to the vehicle‐treated control group and presented as fold change.

### 2.11 In Vivo Study

Forty‐six healthy female volunteers between the ages of 32 and 70 (mean age: 58.3 ± 12.3 years old) were recruited from Gdansk and nearby regions in Poland. Prior to the baseline measurement (D0), all volunteers went through a wash‐out period of 2 weeks, whereby they were instructed to apply a neutral moisturizer instead of their regular facial skin care product. All recruited volunteers were Caucasians of phototype I, II, or III according to Fitzpatrick classification and had various skin types, namely, normal skin (*n* = 3), dry skin (*n* = 25), combination skin (*n* = 17), and oily skin (*n* = 1). Written informed consent was obtained from all the volunteers before enrolment. Measurements were carried out under standard temperature and humidity conditions (22 ± 2°C and relative humidity between 35% and 55%) at baseline (D0), after 28 days (D28), and after 56 days (D56) of twice‐daily product application. The active formulation (containing 1% *G. uralensis* extract) or the vehicle was applied on each hemi‐face according to a randomization in a split‐face study design. The composition of the vehicle formulation is shown in Table 1. The main parameter monitored was the dermal density measured by the DUB SkinScanner from Eotech, at a frequency of 22 MHz. The study was conducted in accordance with the World Medical Association's (WMA) Helsinki Declaration and its amendments (Ethical Principles for Medical Research Involving Human Subjects, adopted by the 18th WMA General Assembly Helsinki, Finland, June 1964, and amendments) and in the spirit of Good Clinical Practices.

**TABLE 1:** Ingredient list of vehicle formulation used during the clinical study. Ingredients are listed in decreasing order from top to bottom.

Vehicle formulation ingredient list
Aqua
Caprylic/capric triglyceride
Cetyl alcohol
Glyceryl stearate
Butylene glycol
Polyacrylate crosspolymer‐6
PEG‐75 stearate
Caprylyl glycol
Ceteth‐20
Steareth‐20
Sodium benzoate
Citric acid

## 3 Results

### 3.1 Data Mined Key Protein–Protein Interactions Mediated by UV and Pollution

**FIGURE 1:**
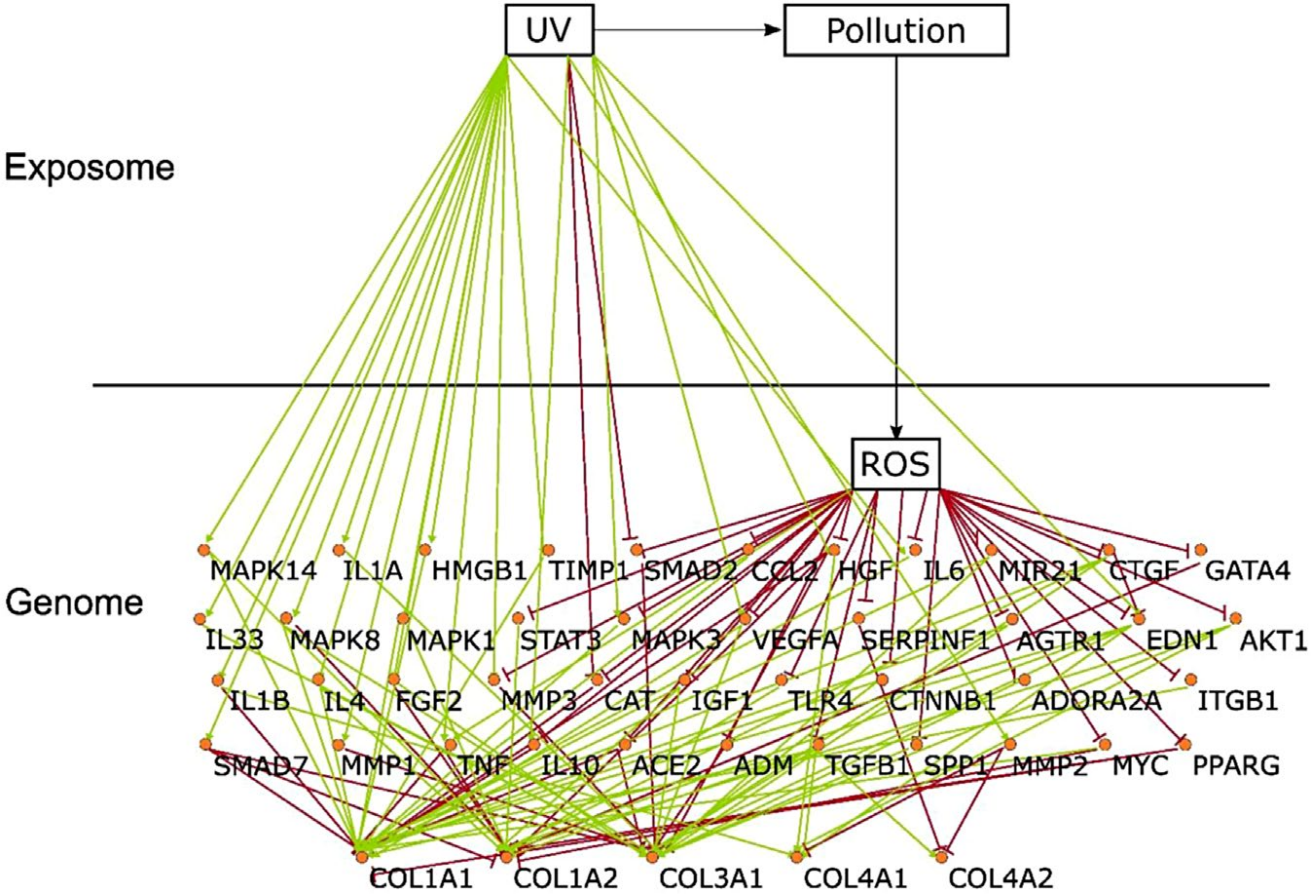
Key interactions between the exposome and the genome and the ROS‐mediated effects of collagens in skin homeostasis. Proteins and their interactions obtained by literature data evaluation of an upstream π–π network for COL1A1, COL1A2, COL3A1, COL4A2 are displayed in the context of human cells and the interaction with UV, ROS, and pollution (DPM).

COL1A1, COL1A2, COL3A1, COL4A1, and COL4A2's interactions mediated by UV, pollution (DPM), and ROS in human skin cells were data mined from the scientific literature. Forty‐one different proteins were identified and their interactions with the exposome and the five different types of collagens are presented in Figure 1.

Next, a manual structure–activity relationship and literature review was carried out, pointing at phytoestrogens such as glycyrrhizin, isoliquiritigenin, liquiritigenin, liquiritin, and glabrol, as promising candidates.

### 3.2 Extract Composition and Stability

The phytochemicals present in the extract were tentatively identified using accurate mass measurement and are listed in Table 2.

**TABLE 2:** List of compounds tentatively identified by HPLC/‐ESI‐QToF in *Glycyrrhiza uralensis* extract.

Phytometabolites	Molecular formula	Theoretical *m/z* [M‐H]^−^	Retention time (min)
Isoviolanthin	C_27_H_30_O_14_	577.1563	13.4
Liquiritin apioside	C_26_H_30_O_13_	549.1614	15.7
Liquiritin	C_21_H_22_O_9_	417.1191	18.2
5‐Hydroxyliquiritin	C_21_H_22_O_10_	433.1140	29.8
Isoliquiritin apioside	C_26_H_30_O_13_	549.1614	32.1
Licorice glycoside D1	C_35_H_36_O_15_	695.1981	34.1
Isoliquiritin	C_21_H_22_O_9_	417.1191	36.8
Liquiritigenin	C_15_H_12_O_4_	255.0663	47.2
Licoricesaponin A3	C_48_H_72_O_21_	983.4493	49.8
Yunganoside K2	C_42_H_62_O_17_	837.3914	54.4
Echinatin	C_16_H_14_O_4_	269.0819	56.6
Licoricesaponin G2 isomer	C_42_H_62_O_17_	837.3914	60.9
Yunganoside G1	C_47_H_74_O_21_	973.4650	61.7
Licoricesaponin G2 isomer	C_42_H_62_O_17_	837.3914	62.6
Yunganoside G2	C_42_H_64_O_17_	839.4071	63.3
Glycyrrhizin isomer	C_42_H_62_O_16_	821.3965	64.9
Formononetin	C_16_H_12_O_4_	267.0663	67.6
Glycyrrhizin isomer	C_42_H_62_O_16_	821.3965	68.9
Uralsaponin	C_42_H_62_O_16_	821.3965	70.3
Licocalchone D	C_21_H_22_O_5_	353.1394	76.3
Licoisoflavone B	C_20_H_16_O_6_	351.0874	78.5
Glycocoumarine	C_21_H_20_O_6_	367.1187	79.9
Gancaonin	C_21_H_20_O_5_	351.1238	82.8

**FIGURE 2:**
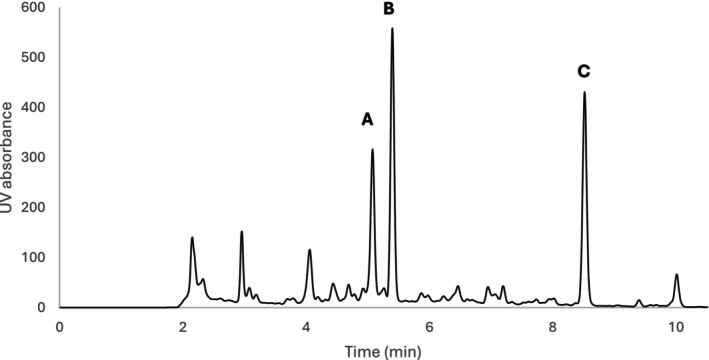
HPLC‐UV chromatogram of a *Glycyrrhiza uralensis* extract used for the quantification of liquiritin apioside (A), liquiritin (B) and liquiritigenin (C).

An extract of *G. uralensis* rhizome was stored over a period of 3 months at three different temperatures and monitored by HPLC‐UV, Figure 2. No significant changes in the quantity of the monitored compounds, liquiritigenin, liquiritin apioside, and liquiritin, were observed at either 4°C or room temperature. However, at 40°C the quantity of liquiritin apioside and liquiritin decreased by (21.7 ± 1.8) % and (46.7 ± 3.3) % respectively, while the concentration of liquiritigenin increased by (62.0 ± 0.7) %.

### 3.3 Cell Viability

No decrease in cell viability was observed in either keratinocytes or fibroblasts after 24 h of treatment with *G. uralensis* extract up to a concentration corresponding to 5 μM liquiritigenin. Therefore, this concentration was selected for use throughout the entire study.

### 3.4 *Glycyrrhiza uralensis* Extract Induces Collagen I and III Gene Expression and Pro Collagen I Protein Expression in Human Primary Skin Cells

**FIGURE 3:**
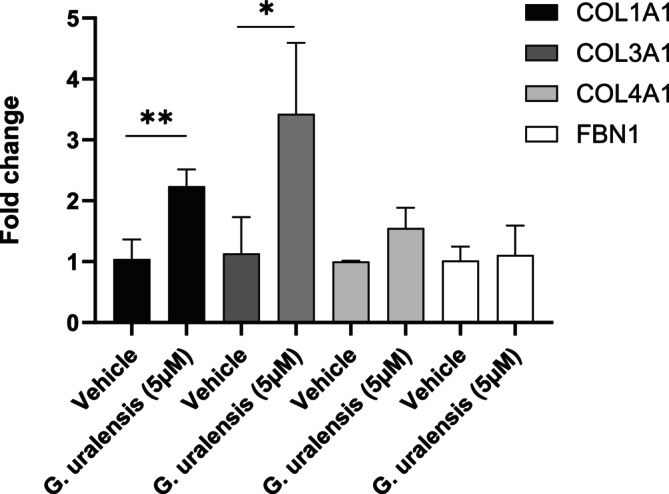
Human dermal fibroblasts were treated for 24 h with *Glycyrrhiza uralensis* extract (equivalent to 5 μM liquiritigenin). Gene expression analysis showed a significant increase of COL1A1, *p*‐value = 0.0018, and COL3A1, *p*‐value = 0.0121. Statistics were performed on delta Ct values using repeated measures one‐way ANOVA, *p ≤ 0.05, **p ≤ 0.01, *n* = 5 in duplicates where *n* is the number of fibroblast donors.

Five fibroblast donors from three experiments were compiled, and the gene expression of Collagen I (COL1A1) and III (COL3A1) was significantly upregulated after treatment with *G. uralensis* extract Figure 3. No significant difference in the gene expression for collagen IV (COL4A1) or fibrillin (FBN1) was observed after treatment with *G. uralensis* compared to vehicle, Figure 3.

**FIGURE 4:**
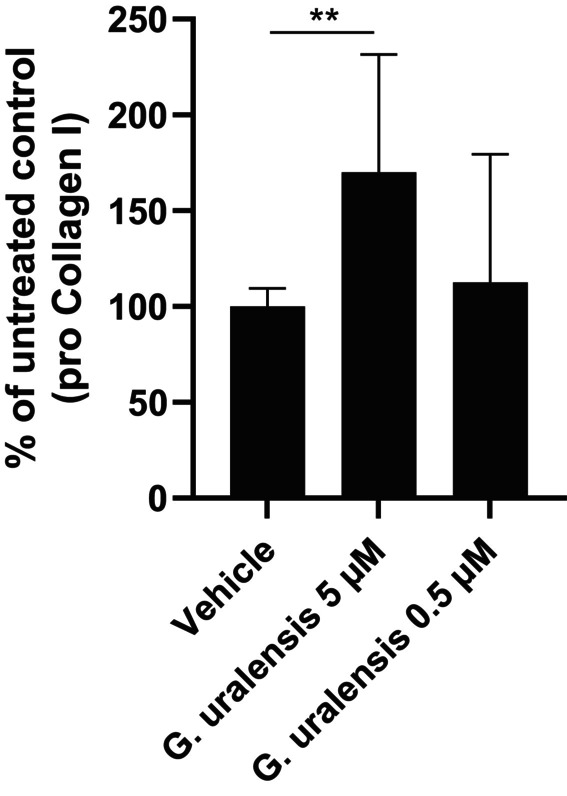
Human dermal fibroblasts were treated for 48 h with *Glycyrrhiza uralensis* extract (equivalent to 5 and 0.5 μM liquiritigenin). Cell media was analyzed and pro‐collagen I concentration is presented as % of untreated control. Statistics were performed on raw data with one‐way ANOVA **p ≤ 0.01, vehicle versus *G. uralensis* 5 μM, *p* = 0.0074. *n* = 6 in duplicates where *n* is the number of fibroblast donors.

Fibroblasts, from 6 different donors, treated with *G. uralensis* extract also resulted in a significant increase of Pro collagen I. In the presence of the highest concentration of extract (corresponding to 5 μM liquiritigenin) a mean of +70% increase compared to the untreated vehicle was observed, Figure 4.

### 3.5 *Glycyrrhiza uralensis* Inhibits UVB Induced MMP1 in Human Primary Fibroblasts

**FIGURE 5:**
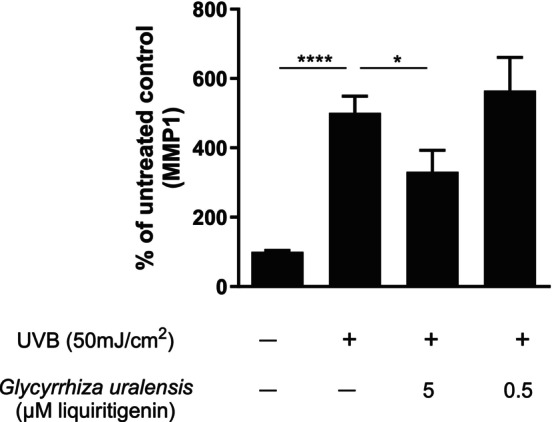
Human dermal fibroblasts were pre‐treated with *Glycyrrhiza uralensis* extract corresponding to liquiritigenin concentration of 5 or 0.5 μM for 1 h and were then treated with the extract in a media from UVB irradiated keratinocytes (50 mJ/cm^2^) for 24 h. MMP1 concentration is presented as a percent of untreated control. Statistics were performed on raw data with one‐way ANOVA *p ≤ 0.05, ****p ≤ 0.0001 UVB versus *G. uralensis* 5 μM treatment, *p* = 0.0213. *n* = 3 in duplicates, where n is the number of fibroblast donors.

Matrix metalloproteinase‐1 (MMP‐1) plays an important role in collagen homeostasis through its degradation of collagen in dermal skin [19]. We therefore examined the effect of *G. uralensis* on UVB‐induced MMP1 expression in human primary fibroblast cells. Significant inhibition of UVB‐induced MMP1 production was shown after 1 h of pretreatment followed by 24 h of treatment with *G. uralensis* in human primary dermal fibroblasts as illustrated in Figure 5. This inhibition is dose‐dependent, with 42% inhibition in the presence of *G. uralensis* extracts corresponding to 5 μM of liquiritigenin.

### 3.6 *Glycyrrhiza uralensis* Inhibits Pollution Induced MMP1 in Human Primary Fibroblasts

**FIGURE 6:**
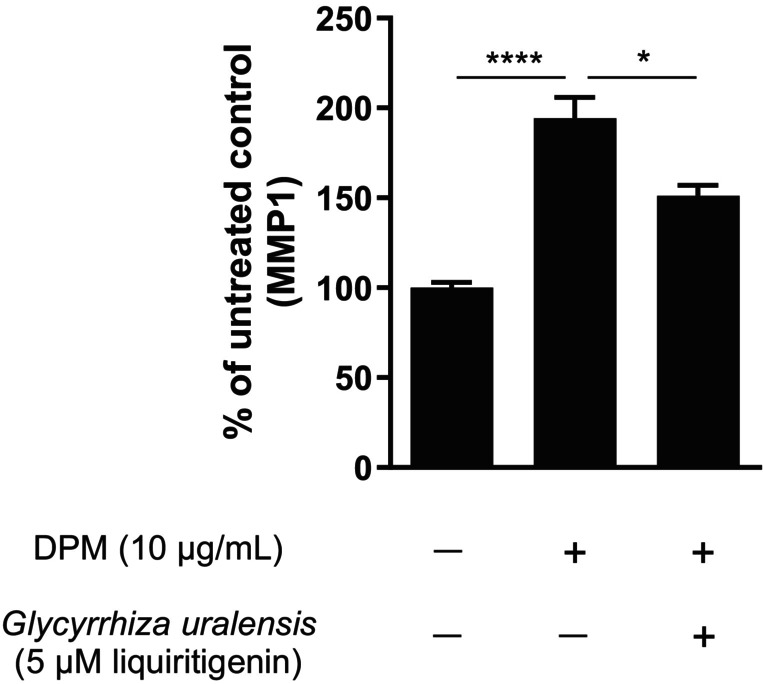
Human dermal fibroblasts pre‐treated with *Glycyrrhiza uralensis* extract (5 μM liquiritigenin) for 1 h, followed by treatment for 24 h with *G. uralensis* extract in media from DPM treated (10 μg/mL) keratinocytes for 24 h. MMP1 concentration is presented as a percentage of untreated control. Statistics were performed on raw data with one‐way ANOVA *p ≤ 0.05, ****p ≤ 0.0001. DPM versus *G. uralensis* 5 μM treatment, *p* = 0.0254. *n* = 3 in duplicates, where *n* is the number of fibroblast donors.

To evaluate the efficacy of *G. uralensis* extract in inhibiting DPM‐induced MMP1 expression, three different fibroblast donors were utilized. The experimental results demonstrated that the *G. uralensis* extract significantly reduced the levels of DPM‐induced MMP1 by approximately 40%, as illustrated in Figure 6.

### 3.7 In Vivo Evaluation of Dermal Density by the DUB SkinScanner

**FIGURE 7:**
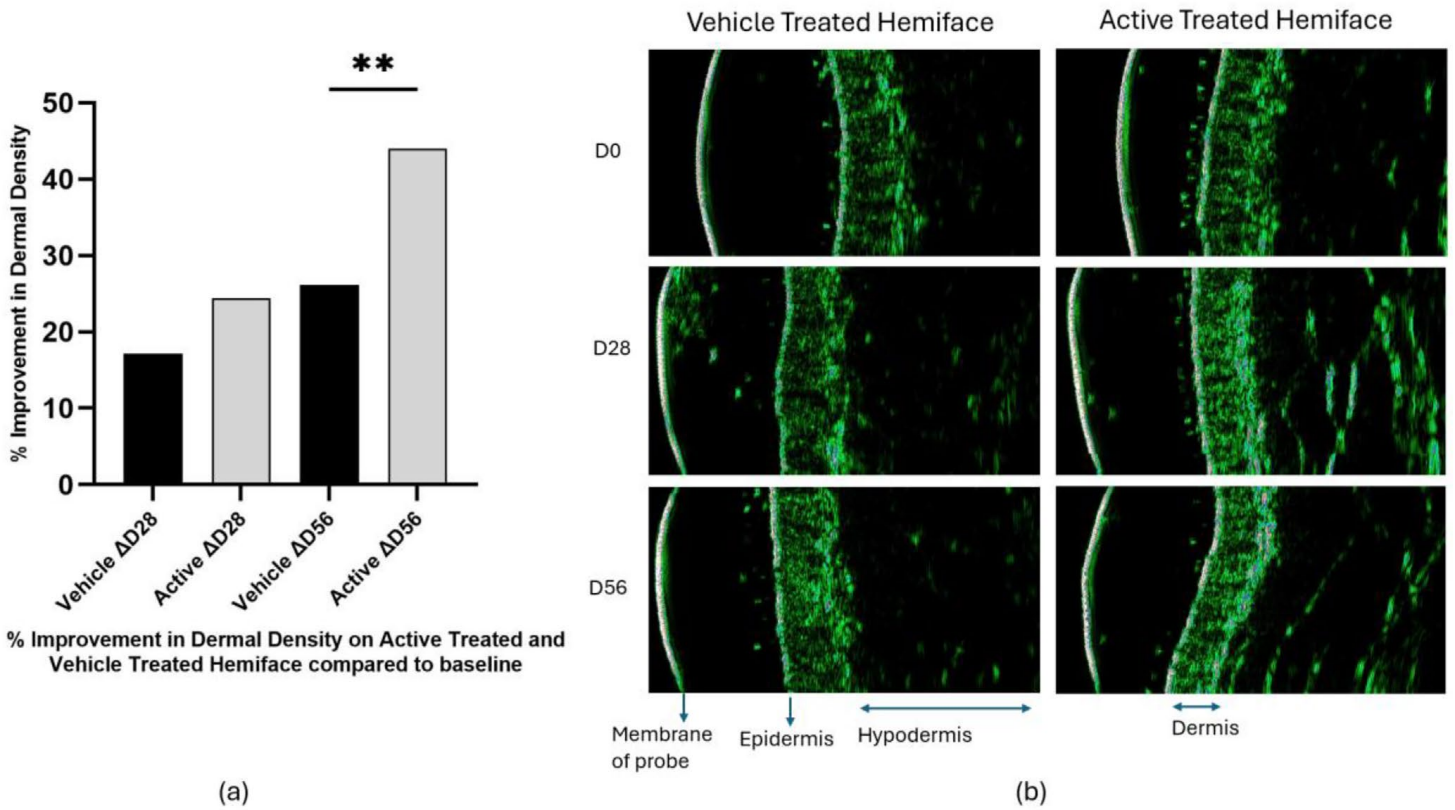
Results from dermal density measurements in an in vivo clinical study. (a) Graph showing percentage improvements of dermal densities of the vehicle‐ and active‐treated hemifaces after 28 and 56 days of twice daily hemiface applications. A paired Student's *t*‐test was performed for statistical analysis where **p < 0.01, vehicle‐ versus active‐treated hemifaces, *p* = 0.0065. (b) Echogenicity images from DUB SkinScanner, show improvement in the skin density of the vehicle‐treated hemiface (left) and active‐treated hemiface (right). The green pixels in the dermal region correspond to the echogenic collagen network.

The DUB SkinScanner was used to measure the changes in the dermal densities of the active‐ and vehicle‐treated hemifaces. Following 28 days of twice daily hemiface applications of the vehicle and active formulations, statistically significant improvements of the dermal densities were observed for both hemifaces compared to baseline (17% for the vehicle and 26% for the active‐treated hemiface), Figure 7a. However, the difference between the active formulation and the vehicle was not statistically significant.

After 56 days of twice daily hemiface application of the vehicle and the active formulation, statistically significant improvements in the dermal densities compared to baseline were observed for both hemifaces (24% for the vehicle and 44% for the active‐treated hemiface), Figure 7a. The superior efficacy of the active formulation compared to the vehicle, in enhancing dermal density, was statistically significant after 56 days of hemiface application, Figure 7b.

As illustrated in Table 3, dividing the participants into two age groups, below and above 50 years old, revealed a difference in the average percentage increase in dermal density compared to baseline between the two groups. In the group with participants > 50 years old, the increase of dermal density for the active‐treated hemiface was 27% at D28 and 49% at D56, while for the vehicle‐treated hemiface, it was only 18% and 24%, respectively. The difference in dermal densities at D56 was statistically significant. In the group with participants < 50 years old, the increase in dermal density of the active‐treated hemiface was 15% at D28 and 30% at D56, while for the vehicle‐treated hemiface, it was 14% and 31%, respectively. The average percentage increases in dermal densities, between the active‐ and vehicle‐treated hemifaces, were not significantly different at any time point.

**TABLE 3:** Percentage improvements in dermal density (compared to baseline) when analyzing sub‐groups based on age.

	Entire studied population		Above 50 years old		Below 50 years old	
	Mean age = 58.3 ± 3 years old *n* = 46		Mean age = 65.1 ± 3.7 years old *n* = 34		Mean age = 38.8 ± 4.4 years old *n* = 12	
	Vehicle	Active	Vehicle	Active	Vehicle	Active
*D28*	17% ± 26%	24% ± 29%	18% ± 27%	27% ± 31%	14% ± 26%	15% ± 17%
*D56*	26% ± 33%**	44% ± 31%**	24% ± 32%**	49% ± 32%**	31% ± 38%	30% ± 23%

** Statistically significant difference between vehicle‐ and active‐treated hemifaces (**p < 0.01, paired Student's *t*‐test), *p* = 0.002.

## 4 Discussion

Estrogen decline, particularly post‐menopause, significantly impacts skin thickness, hydration, elasticity, and wrinkle formation due in part to a significant loss of collagens [2, 20]. This decline requires the implementation of effective interventions to counteract these detrimental effects.

Previous clinical studies have shown that systemic hormone replacement therapy (HRT) and topical estrogen treatments can enhance skin collagen levels and improve skin thickness, elasticity, and hydration [7, 21, 22]. As part of a data mining approach to identify natural compounds that boost collagen for skin care, we identified several phytoestrogens, including liquiritigenin. Liquiritigenin is reported to be the principle phytoestrogen in liquorice extracts [13] and among the liquorice species, *G. uralensis* has been found to possess the highest concentration of liquiritigenin and its glycosides [14, 15]. Therefore, this extract was selected for the study.

The analysis of the *G. uralensis* rhizome water extract revealed the presence of various groups of compounds, with flavonoids, coumarins, triterpenoids, and stilbenoids being the predominant ones, as summarized in Table 2. Quantification of liquiritigenin and its glycosides, specifically liquiritin apioside and liquiritin, indicated that liquiritigenin was the most abundant flavonoid, consistent with previous findings [13]. Liquiritigenin has been used throughout this study as a quantitative chemical marker. Stability analysis of the extract demonstrated that, after 3 months at 40°C, the concentration of liquiritin apioside and liquiritin decreased, while the concentration of liquiritigenin increased. This phenomenon is most likely due to the decomposition of liquiritin apioside and liquiritin to liquiritigenin, as described by Sun et al. [23] The stability of the extract under ambient conditions makes it a promising candidate for topical applications. Additionally, the increased concentration of liquiritigenin at higher temperatures does not negatively affect the extract, as liquiritigenin is the active compound of interest.

Given the presence of multiple phytoestrogens in *G. uralensis* extract and the aim to find natural collagen‐boosting alternatives to HRT and topical estrogens, we investigated the effect of *G. uralensis* extract on collagen induction in human dermal fibroblasts. Our results demonstrated a significant upregulation of gene expression for both Collagen I and Collagen III, as well as pro‐collagen I protein levels, following treatment with *G. uralensis* extract (Figures 3 and 4). These findings align not only with existing literature on other phytoestrogens such as genistein, equol, and daidzein, which have been shown to induce collagen production in the skin but also validate our data mining strategy to identify collagen inducers [11].

The changes in skin structure and function due to estrogen decline also render the skin more vulnerable to external environmental factors. Thinner, less hydrated skin is more susceptible to both UV radiation and pollutants such as particulate matter, polycyclic aromatic hydrocarbons (PAHs), and heavy metals, which can lead to oxidative stress, inflammation, photoaging, and pigmentation changes [24]. Both UV radiation and pollution activate the aryl hydrocarbon receptor (AhR) which leads to an increase in metalloproteinases in the skin, particularly MMP1 [25, 26]. MMP1 is a metalloproteinase responsible for the degradation of collagen in the skin, further exacerbating the decline in collagen levels in estrogen‐deficient skin. In this study, we demonstrated a significant inhibition of both UVB and pollution (DPM) induced MMP1 production after 1 h of pretreatment followed by 24 h of treatment with *G. uralensis* extract in human primary dermal fibroblasts (Figures 5 and 6). A downregulation of MMP1, thereby reducing the degradation of collagen, indirectly results in an increase in collagen in the skin. Previous studies have shown that estrogen and well‐known phytoestrogens such as genistein, equol, and daidzein exhibit both antioxidant and photoprotective effects [27, 28].

The decrease in dermal collagen and elastin, especially after menopause, affects the extracellular matrix and the dermal structure leading to a decrease in both dermal density as well as skin thickness [29, 30]. While the effects of phytoestrogen on collagen are well documented in in vitro and ex vivo studies, most clinical investigations have primarily demonstrated the efficacy of phytoestrogens on the physical aspects of skin aging, such as skin hydration, facial wrinkles, skin firmness, and skin color [21]. However, there are only limited clinical studies demonstrating the efficacy of topical phytoestrogen on dermal structures [31]. Dermal density is a particularly good marker for representing skin health and evaluating the efficacy of cosmetic treatments on skin aging [32].

In the clinical study conducted as part of this research, a cosmetic formulation containing 1% *G. uralensis* extract was compared to a vehicle formulation. Both were applied twice daily for 8 weeks to the hemiface of the entire study population (*n* = 46, mean age = 58 years). A significant increase in dermal density was observed on the hemiface treated with the 1% *G. uralensis* extract compared to the hemi‐face treated with the vehicle formulation, Figure 7. The increase in dermal density correlates well with the rise in dermal collagen I and III gene expression and pro‐collagen I expression observed in the in vitro tests (Figures 3 and 4). This further supports the efficacy of *G. uralensis* extract as an efficient phytoestrogen in a topical formulation for enhancing cutaneous collagen synthesis and combating signs of skin aging in the menopausal population.

Interestingly, a subgroup analysis comparing panelists above the age of 50 (*n* = 34, mean age = 65 years old) to those below 50 (*n* = 12, mean age = 39 years old), revealed that after 8 weeks of product application, the superior efficacy of the active formulation compared to the vehicle was observed only in the estrogen‐depleted population (age ≥ 50). In the above 50 years old population, the average increase in dermal density on the vehicle‐treated hemiface was 27%, while the increase on the active‐treated hemiface was 49% (Table 3). The improvement in the dermal density favoring the active formulation was statistically significant. However, in the population below the age of 50, the average increase in dermal density on the active‐treated hemiface (30%) was comparable to the average increase on the vehicle‐treated hemiface (31%). These results align with the observations of Takuathung et al., where the clinical efficacy of a phytoestrogen was noted in the sub‐group of older women (aged ≥ 50), compared to the younger sub‐group [31]. This difference between the younger and the older subgroups may be explained by the fact that phytoestrogen may behave differently, depending on whether the cutaneous estrogen level is low or high. It has been reported that phytoestrogens may exert their effects as estrogen antagonists in high‐estrogen environments, or they may act as estrogen agonists in low‐estrogen environments [31, 33].

## 5 Conclusions

In conclusion, our data mining approach to identify natural compounds capable of inducing collagen production in primarily estrogen‐deficient skin has been successful. These findings underscore the potential of topical *G. uralensis* extract as a natural alternative to HRT and topical estrogens for enhancing skin health, particularly in post‐menopausal women. The extract not only boosts collagen production in vitro and skin density in vivo, but also offers protection against environmental stressors, making it a multifaceted solution for combating skin aging.

In the future, further improvements can be made by combining our approach with a less biased selection of active compounds, especially given the current progress in quantitative structure‐analysis relationship combined with deep learning. Deep learning has notably been used to find natural compounds regulating targets of interest [34].

## Author Contributions

N.A. and C.O. have contributed equally to this manuscript. Conception and design: L.V.‐J. Acquisition of data: N.A., C.O., J.C., V.L.‐K. and T.R. Analysis and interpretation of data: N.A., C.O., J.C., V.L.‐K. and T.R. Drafting the manuscript: N.A., C.O., J.C. and T.R.

## Ethics Statement

The clinical study was conducted in accordance with the Declaration of Helsinki with all its amendments. Ethical review and approval by the Ethics committee was waived because the study was carried out in Poland and the product under investigation is defined as a cosmetic product according to the European Regulation (EC) No 1223/2009, that is, a product to cleanse, perfume, modify the appearance, protect, maintain the human body in good condition, or to correct body odor. The cosmetic products tested were safe to use, as confirmed by challenge testing and toxicological evaluations. In addition, the guidelines of good clinical practice (GCP) were strictly adhered to during the studies. Informed consent was obtained from all subjects involved in the study.

## Conflicts of Interest

The authors declare no conflicts of interest.

## Data Availability

The data that support the findings of this study are available from the corresponding author upon reasonable request.
